# The influence of the microbiome on radiotherapy and DNA damage responses

**DOI:** 10.3389/fonc.2025.1552750

**Published:** 2025-03-17

**Authors:** Aadil Sheikh, Michael A. Curran

**Affiliations:** ^1^ Department of Medical Education, Foster School of Medicine, Texas Tech University Health Sciences Center El Paso, El Paso, TX, United States; ^2^ Department of Immunology, The University of Texas MD Anderson Cancer Center, Houston, TX, United States

**Keywords:** radiotherapy, microbiome, colorectal cancer, DNA damage, DNA repair

## Abstract

Colorectal cancer (CRC) is one of the most prevalent cancers in terms of diagnosis and mortality. Radiotherapy (RT) remains a mainstay of CRC therapy. As RT relies on DNA damage to promote tumor cell death, the activity of cellular DNA damage repair pathways can modulate cancer sensitivity to therapy. The gut microbiome has been shown to influence intestinal health and is independently associated with CRC development, treatment responses and outcomes. The microbiome can also modulate responses to CRC RT through various mechanisms such as community structure, toxins and metabolites. In this review we explore the use of RT in the treatment of CRC and the molecular factors that influence treatment outcomes. We also discuss how the microbiome can promote radiosensitivity versus radioprotection to modulate RT outcomes in CRC. Understanding the molecular interaction between the microbiome and DNA repair pathways can assist with predicting responses to RT. Once described, these connections between the microbiome and RT response can also be used to identify actionable targets for therapeutic development.

## Introduction

Colorectal cancer (CRC) is the third most commonly diagnosed cancer and the third leading cause of cancer-related mortality in the United States and globally (1, 2). While the incidence of CRC differs between regions, there is a strong correlation between incidence and level of socioeconomic development with a majority of cases being concentrated in North America, Western Europe and Australia (3, 4). Many CRC cases are strongly associated with modifiable risk factors including environment, lifestyle and diet (5, 6) and advancements in screening and treatment options have helped to mitigate some of this disease burden (7–9). Despite these advances increasing exposure to risk factors such as chronic health conditions including obesity and metabolic syndromes that promote long-term low-grade inflammation and the increasing adoption of the metabolically challenging “Western diet” is leading to rising case numbers and increasing incidence amongst young adults, demonstrating the major challenge that CRC poses to long-term public health and safety (7–9).

**Figure 1:**
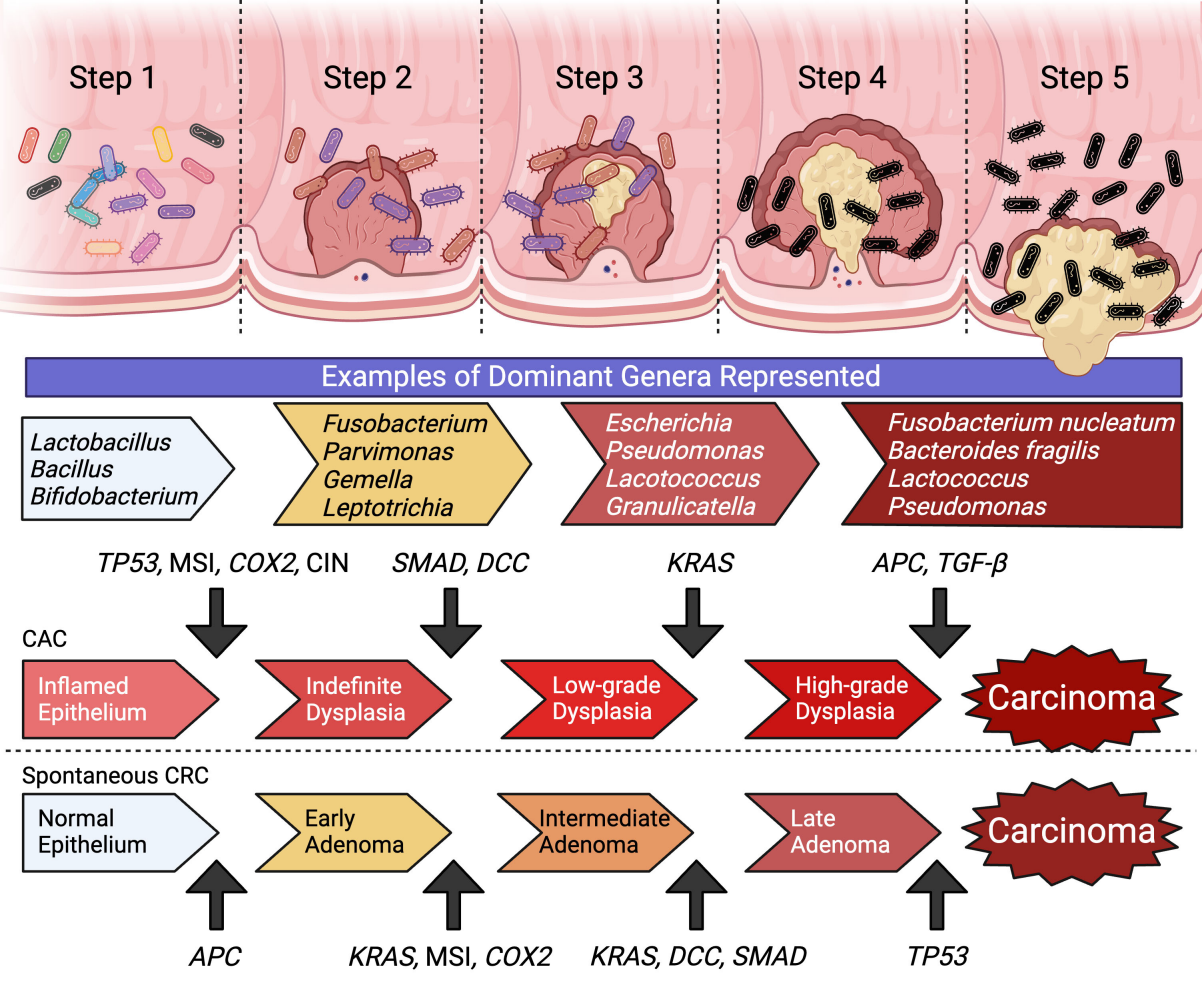
The molecular carcinogenesis of spontaneous CRC and CAC represent two distinct pathways of step-wise accumulation of mutations. Within the development of colon cancer there is associated with the enrichment of specific microbial genera and a concurrent loss of diversity and community structure. (Created with BioRender.com).

The molecular pathway of colorectal carcinogenesis is well documented with the step-wise accumulation of point mutations in critical genes that correlate to morphological changes beginning with adenoma and ending with carcinoma (10–12). Sporadic CRCs constitute a majority of the cancer cases, typically beginning with a mutation in the adenomatous polyposis coli (*APC*) gene, with subsequent mutations in *KRAS, SMAD4, TP53* and *DCC* (10–12) (**Figure 1**). Inherited CRC form a smaller proportion of reported cases and include familial adenomatous polyposis (FAP) and Lynch syndrome (13, 14). These disease are associated with specific heterozygous inactivating mutations in *APC* and mismatch repair genes (i.e: *MLH1, MSH2, MSH6*) respectively (13–16). A sub-population of CRC cases that has been increasing in recent years is associated with chronic inflammatory states such as inflammatory bowel diseases (IBDs) (17, 18). These colitis-associated CRCs (CACs) are associated with earlier presentation, less detectable lesions, and increased genomic instability and mutational burden (17, 19–21). CACs have molecular pathogenesis that is distinct from sporadic CRCs, typically beginning with a mutation in *TP53*, and subsequent mutations in *KRAS* and *SMAD4* (22) (**Figure 1**). An important contributor of intestinal inflammation is the microbiome and microbial community structure (23–25). Disruption of the symbiotic interactions of the microbiome with host epithelial and immune cells and/or development of dysbiosis can lead to chronic inflammation and pre-dispose patients to developing CAC (23–25).

The microbiome is highly personalized and can influence responses to cancer treatment modalities and outcomes (26, 27). Carcinogenesis in the intestinal tract is associated with unique microbial community signatures. Early stages are associated with genera of *Fusobacterium, Parvimonas, Gemella* and *Leptotrichia* with typical lesions showing enrichment of *Escherichia coli* and *Pseudomonas veronii*, and ~40-60% of carcinomas having high carriage of *Fusobacterium* (**Figure 1**) (28, 29). The metabolism of resident microbes can improve cancer susceptibility to treatment or potentially confer resistance by modifying efficacy and toxicity of different treatment options (30). Additionally, each different class of CRC therapy can uniquely remodel the gut microbiota to influence patient outcomes (31). The effects of the microbiome on chemotherapy and immunotherapy has been studied, however data on the microbial interactions with radiotherapy (RT) is limited (30, 31). This review explores the contribution of the microbiome to the mechanism of action and responses to RT, and how this interaction may be modulated as a novel therapeutic target to improve patient outcomes.

## Radiotherapy and colorectal cancer: usage, mechanisms and outcomes

Radiotherapy is a critical modality for cancer treatment that has been in use for decades (32). The rationale for RT is to utilize focused ionizing radiation on rapidly proliferating cancer cells leading to senescence and cell death (33, 34). RT can be delivered through several methods, either as an external beam delivered via a targeting device (external beam radiation therapy), through the placement of radioactive material near the site of the tumor/lesions (brachytherapy), or through systemic hematological dissemination of radioactive substances such as iodine or radioconjugated antibodies or pharmaceuticals (radioligand therapy) (35).

**Figure 2:**
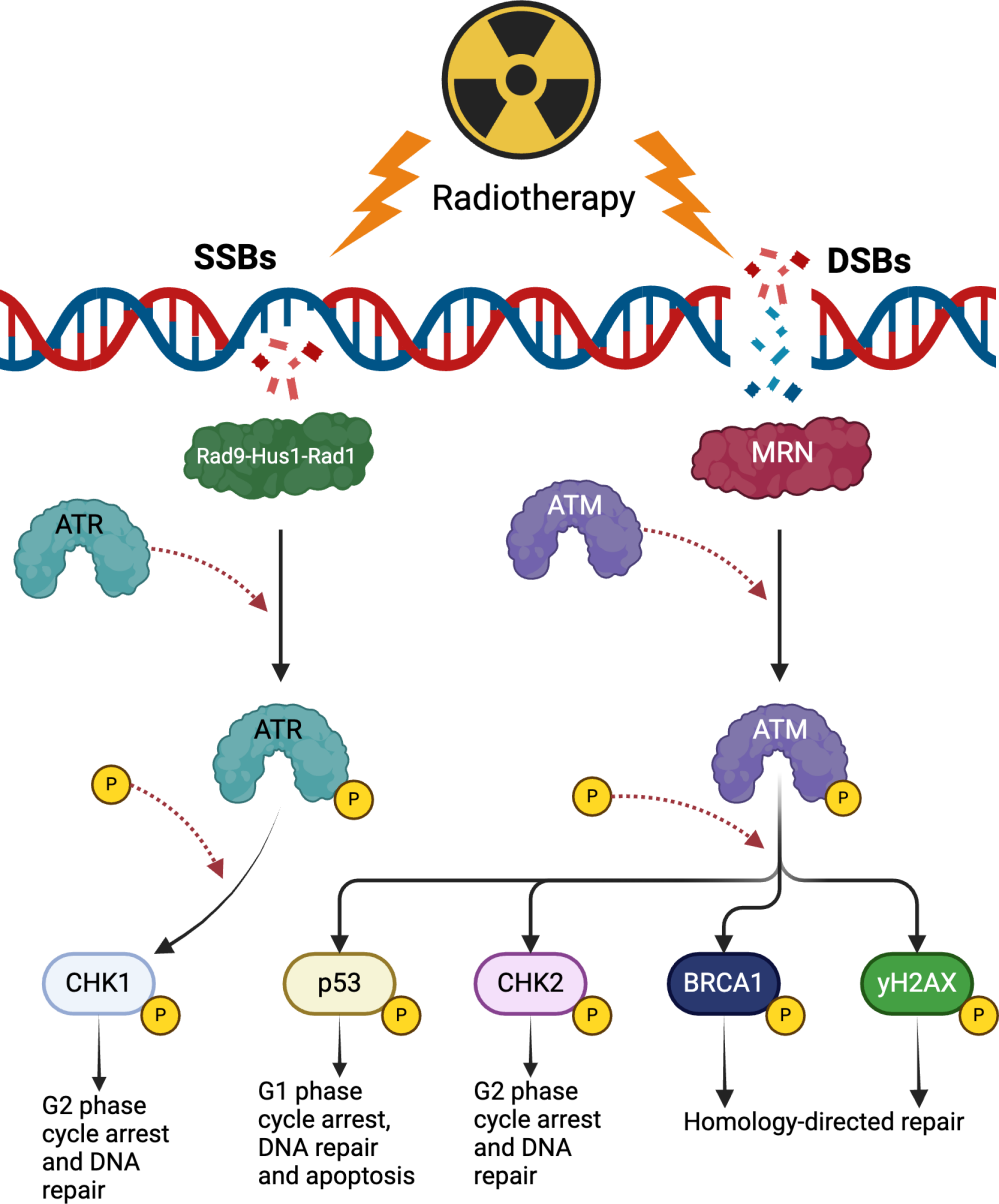
The effects of radiotherapy on DNA damage repair. Radiotherapy produces single-stranded breaks (SSBs) and double-stranded breaks (DSBs). SSBs are detected by the Rad9-Hus1-Rad1 complex that subsequently recruits and activates ATR. ATR phosphorylates CHK1 to promote arrest of the cell cycle in the G2 phase and activate appropriate DNA repair pathways. DSBs activate ATM which phosphorylates multiple substrates such as p53, CHK2 and BRCA1 to promote cell arrest, DNA repair and apoptosis. (Created with BioRender.com).

Radiation promotes several types of DNA damage in target cells including double-stranded and single-stranded breaks (DSBs and SSBs), base damage and inter-strand cross-links (36). A majority of radiation-induced cell death can be attributed to unrepaired DSBs which promote cell cycle arrest, genomic instability and mitotic catastrophe (34, 37). To ensure survival, cancer cells exploit DNA repair pathways to abrogate the damage caused by radiation. The first factor to localize to DSBs is the Mre11-Rad50-Nbs1 (MRN) complex which promotes rapid recruitment and activation of the kinase Ataxia Telangiectasia Mutated (ATM) via phosphorylation (38–40). Activation of ATM results in the downstream phosphorylation of many targets including p53, CHK2, H2AX and BRCA1 to promote cell cycle arrest and the activation of homologous and non-homologous end-joining repair mechanisms (**Figure 2**) (38–40). Another sentinel of DNA damage that works in conjunction with ATM is ATM- and Rad3 Related (ATR) which senses a wider range of DNA damage patterns including DSBs and SSBs. ATR is recruited to the site of lesions by the Rad9-Hus1-Rad1 complex and promotes the phosphorylation of CHK1 along with the subsequent activation of downstream repair mechanisms (**Figure 2**) (36, 39).

In addition to direct cell killing via DNA damage, RT can promote immunogenic cell death (ICD) that results from the release of factors that stimulate the immune system to mount an anti-cancer response through various mechanisms. The innate immune system has evolved multiple receptors to sense ligands released by stressed and damaged cells collectively known as damage-associated molecular patterns (DAMPs) that are generated as a consequence of RT. There are three components of ICD that promote anti-tumor activity by the immune system: the release of ATP into the extracellular space where it acts as a chemoattractant and activator of antigen-presenting cell (APC) inflammasomes via interactions with the P2X receptors (41–43). The second component is the extracellular release of HMGB1 from the nucleus and its subsequent binding to TLR4 which promotes the activation of APCs and antigen-presentation to T cells (34, 44, 45). The final arm of ICD is the presentation of calreticulin on the outer leaflet of the cell membrane, providing an “eat me” signal to APCs and promoting phagocytosis of the dying cancer cells (34, 45). These three pillars of ICD are induced by RT and trigger the release of tumor-associated antigens (TAAs) which are taken up by APCs. This, in turn, produces an anti-tumor vaccination effect via cross-presentation, thereby promoting a robust systemic immune response utilizing both the innate and adaptive arms of anti-tumor immunity (34). While ICD can enhance radiosensitivity of cancer cells through its “tumor vaccine” immune effect, the activation of DNA repair pathways post-RT can prove detrimental to treatment outcomes by promoting radioresistance. The activity of ATM and ATR in response to RT can promote the up-regulation of “don’t eat me” signals in CRC cells such as PD-L1 and CD47 (46). The upregulation of such ligands promotes higher rates of cancer immune escape and proves to be a promising target for neo-adjuvant therapy of CRC (46).

Radiotherapy has been a standard of care for CRC for nearly thirty years (47). While it is more commonly utilized to treat rectal cancers, this modality has also been used to treat colon cancer patients at various stages of their therapeutic course, though the guidelines for utilizing RT in this context are less well defined (48, 49). For these patients, RT can be used in the neo-adjuvant setting in combination with chemotherapy, during or post-surgery (adjuvant) for removing residual cancer cells, or to treat challenging CRC cases where more conventional treatment methods are not feasible (48–53). Despite the advancements made in CRC therapeutic development, the influence of the microbiome on responses and outcomes to RT has not been fully elucidated. Understanding the interactions of the microbiome with different treatment modalities can improve their efficacy and personalize treatment options to a patient’s specific needs potentially yielding improved responses. The gut microbiome plays a crucial role in health and disease, and significant gains have been made to understand its interactions with chemotherapy (30, 31). However, its interactions with RT are still being determined and represent a promising new frontier for cancer research.

## The interactions of radiotherapy and the microbiome

**Figure 3:**
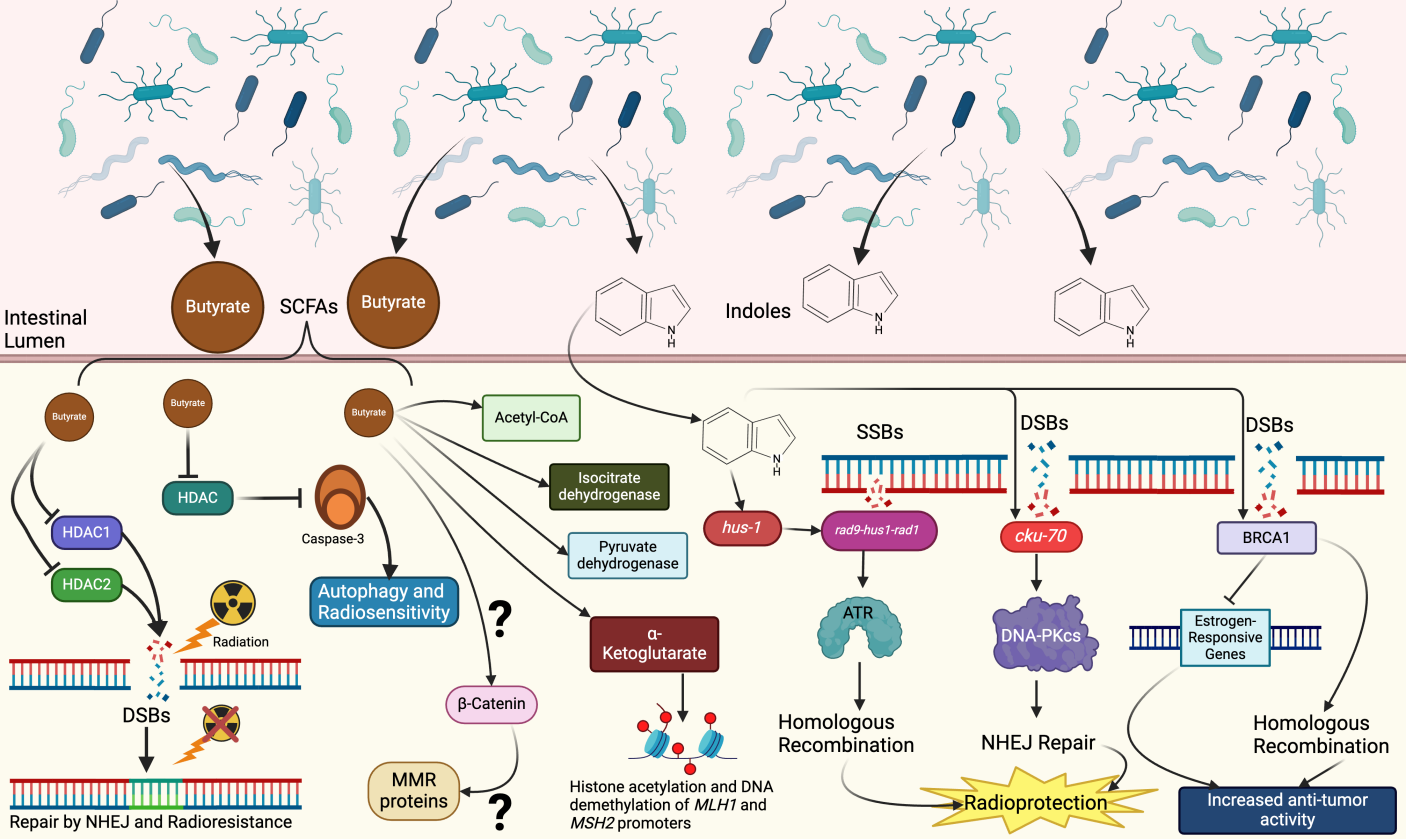
The influence of radiation therapy on microbial community structure. Prior to RT patient fecal matter data demonstrates high species richness within (alpha diversity) and across (beta diversity) patients. Generas represented in these samples are enriched in *Lactobacillus, Bacillus, Bifidobacterium*, and other commensal microbes. Post-RT there is a marked decrease in community richness and structure within 16 hours. Additionally, there is a observed decrease in the *Lactobacillus, Bifidobacterium and Enterobacteriaceae* and an increase in pathogenic species belonging to Fusobacterium and Proteobacteria. (Created with BioRender.com).

While RT has demonstrated significant efficacy in the management and treatment of CRCs, and advancements have allowed for increased precision during treatment delivery, it’s application remains limited by adverse effects on normal tissue (54). Specifically, the production of reactive-oxygen species (ROS) and DNA damage during RT effects the surrounding normal gastrointestinal epithelium, leading radiation-induced enteritis (55). The deleterious effects of RT also extend to the microbiome. RT can potentially influence microbial community structure through the elimination of commensal bacteria, leading undesirable dysbiosis due to the proliferation of pathogenic species that can further promote negative health outcomes (27, 55, 56). An exploratory analysis of 11 patients with pelvic cancers (i.e. CRC and cervical cancer) who developed adverse symptoms after RT demonstrated a lower alpha diversity index, species richness and an increased *Firmicutes/Bacteroidetes* ratio prior to RT when compared to healthy controls (57). Analysis of the patient microbiota post-RT demonstrated significant alterations to the differences with patients that developed diarrhea having unique microbial species profiles compared to patients who did not develop adverse symptoms (57). Further analysis of patient fecal samples over the course of treatment demonstrated a gradual decrease in species diversity in patients who experienced radiation enteropathy (54, 58). Johnson et al. observed a significant decrease in *Lactobacillus, Enterobacteriaceae*, and aerobic bacteria counts within 16 hours of RT (**Figure 3**) (59, 60). Additionally, a systematic review of 11 studies demonstrated an increase in pathogenic bacteria belonging to Proteobacteria and Fusobacteria alongside a significant decrease in commensals such as *Faecalibaterium* and *Bifidobacterium* (**Figure 3**) (54, 61).

Dysregulation of the microbiota and decreased diversity is associated with colitis and IBD. Fecal matter analysis of radiation-treated mice demonstrated an increase in beta diversity when compared to controls and an abundance of opportunistic bacteria from *Akkermansia, Bacteroides*, and *Proteobacteria* (62). Fecal matter transplantation (FMT) of the radiation-treated microbiota into germ-free (GF) mice rendered them more susceptible to radiation injury and DSS-induced colitis, and was associated with increased levels of the inflammatory cytokines IL-1β and TNF-α when compared to naïve microbiomes (62). Treatment with IL-1 receptor antagonists reduced post-radiation cytokine expression and colonic damage (62). Together the data suggests that radiation can influence the composition and diversity of the patient microbiome. The proliferation of obligate and opportunistic pathogens and the simultaneous depletion of commensal and probiotic taxa suggest that the microbiome and RT can have a synergistic effect that increases the chances of developing adverse outcomes. Concurrently the findings also highlight promising new therapeutic targets to ameliorate symptoms such as blockade of inflammatory cascades or probiotic supplementation. Patients that were provided with probiotics such as *Lactobacillus* and *Bifidobacterium* prior to radiation were found to have lower rates of diarrhea and enteritis compared to placebo groups suggesting that preventive intervention to minimize RT induced dysbiosis is feasible (63).

Given the impacts of RT-induced dysbiosis on patient outcomes, there has been a growing interest in exploring how to manipulate the microbiome to improve the efficacy of therapy and to reduce adverse consequences. Studies demonstrate that the microbiome can mitigate the negative effects of RT and even promote cellular radiosensitivity to improve clinical results (55). A seminal study reported finding a significant radioprotective potential of the microbiome and microbial-derived metabolites such as short-chain fatty acids (SCFAs) and indoles. Researchers found a small percentage of mice that could survive high doses of radiation and maintain a normal lifespan (55, 64). The mice were designated as “elite survivors” and were found to have a gut microbiome enriched with *Lachnospiraceae* and *Enterococcaceae*. FMT of elite survivors into GF and specific-pathogen free (SPF) mice demonstrated an increase in survival rate of recipients post-radiation (64). Analysis of fecal samples from patients with leukemia corroborated the findings of the murine study; patients who did not experience diarrhea post-radiation had an abundance of *Lachnospiraceae* and *Enerococcaceae* (64). Further investigation to understand the correlation between radioprotection and *Lachnospiraceae* revealed that SCFAs like acetate, butyrate and propionate produced by the bacteria can mediate radioprotection through inhibition of inflammatory pathways, reduction of ROS generation in cells, and decreased DNA damage (64).

In addition to protecting patients from the negative consequences of RT, the microbiome has demonstrated a capacity to improve the efficacy of radiation. Treatment of murine cancer models with antibiotics to deplete the microbiome prior to radiation has been shown to reduce its efficacy while simultaneously expanding commensal fungi populations (65). Depletion of the commensal fungi and re-establishment of bacteria resulted in a decrease in the count of pro-tumor macrophages and an increase in anti-tumor CD8+ T cell mediated responses (65). Analysis of the microbiota of patients that responded to RT showed an enrichment of *Faecalibaterium* when compared to patients that did not respond to treatment (66). Further metabolomics revealed that cyclic-di-AMP, a second messenger produced by the bacteria, is upregulated in responsive patients and activates the cGAS-STING pathway to regulate immune sensitivity to RT (66). Microbial metabolites such butyrate and methylglyoxal can also promote radiosensitivity through activation of innate immune receptors, and autophagy/apoptosis (67, 68). The emerging data suggests that the manipulation of the microbiome through targeted antibiotics, supplementation with probiotics, or FMT can improve RT outcomes (63–65). While the exact mechanisms governing the interactions of the microbiome and radiosensitivity have yet to be revealed, there are promising results that point to activation of the immune system and DNA damage responses that correlate with the mechanism of action of RT.

## DNA damage repair and the microbiome

With increasing evidence demonstrating the interplay of the microbiome with outcomes from RT, it has become imperative to understand the mechanisms that govern these interactions. While the microbiome affects many physiological processes, in the context of RT it is critical to elucidate the influence of the microbiome on DNA repair pathways. As RT relies on DNA damage to induce cell death, the subsequent activation of DNA repair can promote cancer cell survival and lead to poor therapeutic outcomes (32, 46).

**Figure 4:**
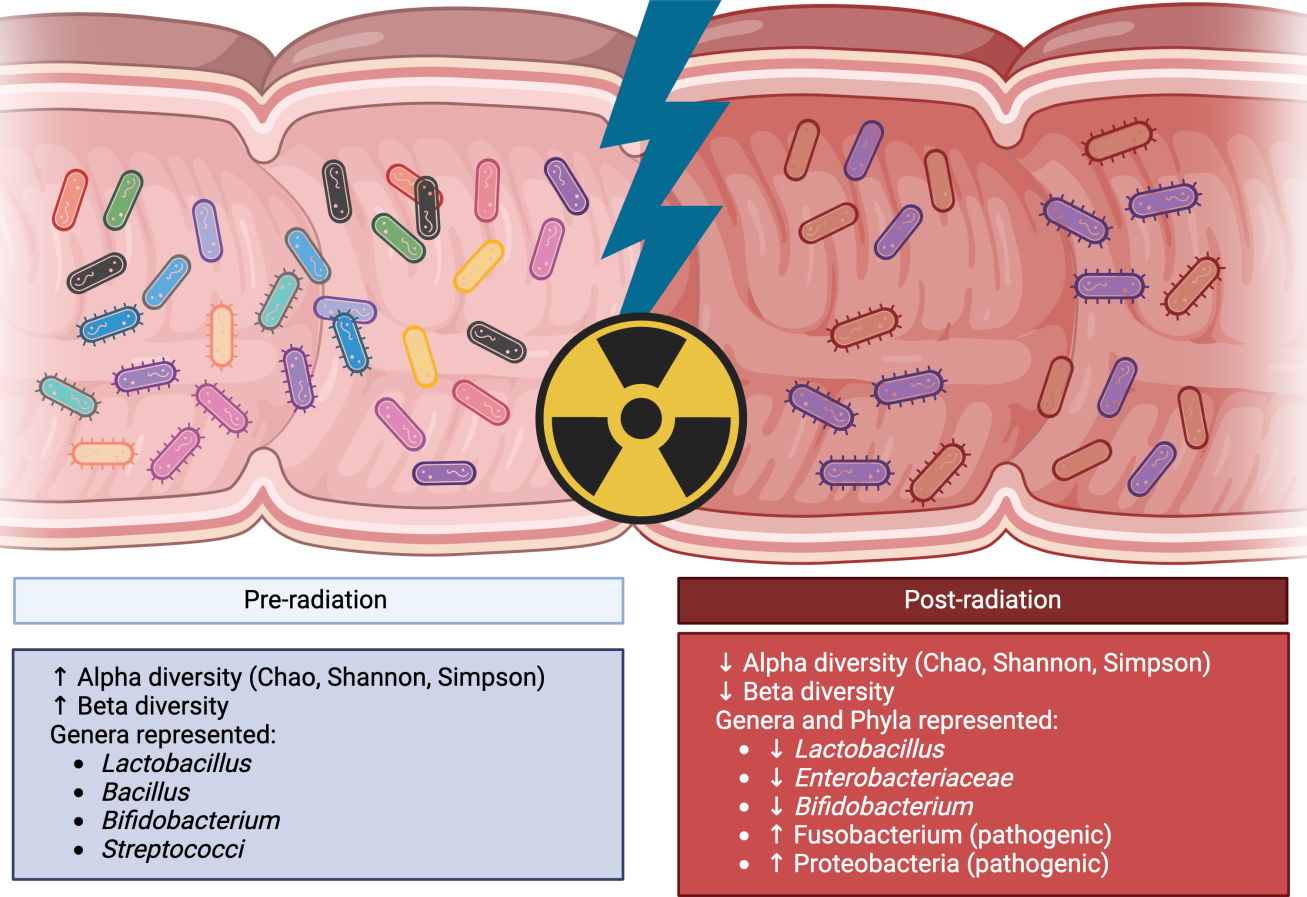
Potential mechanisms by which the microbiome can influence RT responses through interactions with DNA repair pathways. Butyrate and other SCFA products from fermentation of fiber in the intestinal lumen can promote DNA repair through multiple pathways. Butyrate-mediated HDAC inhibition can prompt DSB repair through NHEJ and contribute to radioresistance. Simultaneously it can promote activation of caspase-3 via HDACi which leads to autophagy and radiosenstivity. Butryrate also upregulates the expression of isocitrate dehydrogenase, pyruvate dehydrogenase, ACoA and α-Ketoglutarate. Interaction of butyrate with α-Ketoglutarate leads to epigenetic remodeling of the *MLH1* and *MSH2* promoters which suppress CRC development and can potentially radiosensitize cells. Butryrate can interact with β-catenin through an unknown mechanism independent of APC which in turn has an unknown interaction with MMR proteins to drive proliferation in dMMR CRC. Indole metabolites from the microbiome can induce similar opposing effects as SCFAs. Indoles can interact with repair proteins like *hus-1* to promote homologous repair and *cku-70* to promote NHEJ. Activation of both pathways have been shown to have radioprotective effects in *C. elegans* embryos. Indoles can also work with BRCA1 to inhibit expression of estrogen-responsive genes in breast cancer. Further investigation into the role of indole-DNA repair interactions in CRC can further understanding of RT outcomes and responses. (Created with BioRender.com).

Multiple studies have demonstrated a correlation between the microbiome and cancer-inducing DNA damage in CRC, particularly with in relation to specific species such as *Bacteroides fragilis* and *Fusobacterium nucleatum* (69–72). However, limited data exists on the specific pathways bridging the microbiome and alterations in DNA repair, though there is compelling evidence suggesting a potential link through SCFAs and other microbial-derived metabolites in influencing DNA repair activity (26). SCFAs originate from the metabolism of non-dietary carbohydrates by the microbiome and have many documented effects on health and homeostasis (73). Butyrate has been shown to have cytotoxic effects on CRC cells through the inhibition of histone deacetylases (HDACs) which can promote apoptosis (**Figure 4**) (26, 74, 75). HDACs regulate epigenetic modification of histones through removal acetyl groups resulting in chromatin condensation and transcriptional repression. This HDAC-mediated modulation of gene expression affects carcinogenesis and radioresistance by influencing DNA repair, apoptosis and cell cycle pathways (76, 77). HDACs are upregulated in CRC and are a promising therapeutic target to improve treatment efficacy and outcomes (78–80). HDAC1 and HDAC2 are enriched in CRC cells and have promote radioresistance by localizing to DSBs and activate DNA repair through non-homologous end-joining (NHEJ) (81). Inhibition of HDACs in CRC has demonstrated increased apoptosis and reduced cell growth; further investigations show that HDACi improves CRC radiosensitivity through induced expression of Caspase-3 and stimulation of autophagy (82, 83). Other metabolites originating from commensal bacteria have been found to have radioprotective effects on cells through several DNA repair pathways (84). Indoles produced by commensal *E. coli* have been shown to prevent DNA damage and aneuploidy in *Caenorhabditis elegans* by interacting with *hus-1* and *cku-70*, components of SSB and DSB repair pathways respectively (**Figure 4**) (84). Within the context of cancer, it has been found that indole-3-carbinol (I3C), a natural compound from vegetables, has significant anti-tumor properties in breast cancer (85). I3C has been shown to down-regulate the expression of estrogen-responsive genes and upregulate and work synergistically with BRCA1 to suppress estrogen receptor signaling (**Figure 4**) (85). Though the evidence demonstrates the influence of indoles on DNA damage detection and repair, the precise mechanism by which these molecules dynamically influence radioprotection or radiosensitivity have yet to be fully elucidated.

A prominent subset of CRC is defined by mismatch repair (MMR) deficiencies, characterized by a high frequency of microsatellite instability (86). This characteristic is high penetrant in hereditary syndromes such as Lynch syndrome (hereditary nonpolyposis CRC; HNPCC) due to germline mutation in MMR genes such as *MLH1, MSH2*, and *MSH6* (86). Patients with CRCs that are MMR deficient (dMMR) are found to have higher tumor mutational burden (TMB) and respond to immunotherapy better than patients MMR-proficient (pMMR) CRC (87). The differences in clinical and biological characteristics between dMMR and pMMR cancers extend to the microbiome (87). Studies have demonstrated an enrichment of species that have a high association with CRC such as *Bacteroides fragilis* and *Fusobacterium nucleatum* in patients with dMMR CRC (88). Additionally, it has been found that patients with pMMR CRC tended to have higher alpha diversity and distinct beta diversity when compared to dMMR CRC patients, with the latter having enrichment in genera such as *Fusobacterium, Streptococcus, Akkermansia*, and *Prevotella* (89). A study analyzing the development of CRC in *MSH2*-deficient mice showed that antibiotic depletion of the microbiome prevented the development of polyps (90). Further metabolic analysis implicated butyrate in promoting higher cell turnover and increased differentiation in transit-amplifying cells in the colonic crypts (75, 90). The authors of the study hypothesized that butyrate drives proliferation and increased β-catenin activity in dMMR colon cells (90). They found that that β-catenin is increased *MSH2*-deficient cells in an APC-independent manner and postulated that MMR proteins can directly affect β-catenin signaling, though the exact mechanism remains to be elucidated (**Figure 4**) (90). Paradoxically it has also been found that butyrate can suppress CRC development through MMR via epigenetic remodeling (75). In HT-29 and Caco-2 cells, butyrate has been found to upregulate expression of isocitrate dehydrogenase and pyruvate dehydrogenase, as well as increase levels of acetyl-CoA and α-ketoglutarate (75). Furthermore, butyrate was found to interact with α-ketoglutarate to promote histone acetylation and DNA demethylation of the promoters of *MLH1* and *MSH2* (**Figure 4**) (75). Consideration of MMR-status is becoming more important in CRC treatments due to its role as a molecular biomarker and prognostic indicator (91). While dMMR patients have been shown to have improved disease-free survival with neo-adjuvant chemotherapy, it has also been shown that dMMR tumors respond poorly to RT while pMMR patients respond well (91).

While the mechanistic associations remain to be described, the sum of the existing data suggests a compelling association between the microbiome and microbial metabolites and susceptibility to RT that at least partially depends on modulation of DNA damage repair pathways. Future studies which elucidate the pathways connecting specific microbial elements to their cognate mechanisms of modulation of RT sensitivity may open new avenues for therapeutic intervention to increase RT efficacy while diminishing adverse effects on normal tissue via targeted microbiome modulation. While some therapeutically actionable microbial pathways are likely widely shared, other interventions may require more personalized microbiome profiling and customized therapeutic interventions.

## Conclusions

The microbiome has well-documented effects on host physiology, carcinogenesis and treatment outcomes (25, 27, 28, 56, 88). While the interactions of the microbiome with chemotherapy in CRC are well-appreciated, less work has been done to understand the interplay of the microbiome with RT (26, 31). Though RT is not as common a treatment modality for colon cancer as it is for rectal cancer, it is becoming increasingly used for CRC as evidence emerges for its efficacy in treating locally advanced tumors (32, 51). As RT utilizes DNA damage to promote cell death and anti-tumor responses, it is evident that factors enhancing or dampening responses need to be considered to maximize the efficacy of treatment (30, 32, 33, 43, 51). Microbial community structure, colonization of specific genera, and metabolites have demonstrated a capacity to promote radiosensitivity or, in some cases, radioresistance in CRC (27, 31, 54). Given the microbiome’s capacity to manipulate multiple molecular pathways within host cells, an important question arises as to how it influences DNA repair pathways in CRC and, as a result, modulates RT effectiveness (58, 67, 75). Several studies have investigated the mechanisms involving the microbiome and DNA repair. SCFAs such as butyrate have been demonstrated to have contradictory effects on DNA repair. On one hand it has been shown to upregulate NHEJ by inhibiting HDACs, and can inhibit cancer progression by promoting activation of silenced MMR genes through DNA demethylation and histone acetylation in an α-ketoglutarate dependent manner (26, 75). Conversely, butyrate can promote oncogenic progression and exacerbate dMMR CRC by interacting with β-catenin independent of APC (90). Other metabolites such as indoles can promote radioprotection by interacting with proteins in SSB and DSB repair pathways such as BRCA1 (84, 85). Investigating the impact of the microbiome on DNA repair pathways directly informs on the downstream sensitivity to RT and warrants further investigation.

## Future directions

Several future research directions may be pursued in the future and will help us understand the molecular mechanisms that influence the microbiome-DNA repair-radiotherapy axis. The first will be to investigate the association of microbial community structure and specific species with the expression of various DNA repair proteins involved in NHEJ, homologous end-joining (HEJ), base excision repair (BER), and nucleotide excision repair (NER) in CRC cells. Additionally, it will be critical to define the role of specific microbial metabolites, proteins, or even toxins to influence the different repair pathways either through direct or epigenetic interactions. Furthermore, understanding how these DNA repair proteins will be modified in the presence of a combination of microbial products and RT will assist with understanding key therapeutic windows for dosage and length of treatment to maximize efficiency when moving these strategies to the clinic. Also considering the influence of different methods that impact the microbiome such as probiotics, prebiotics, antibiotic and FMT will provide evidence that can inform standard-of-care guidelines. Such advances will enable us to establish a direct mechanistic link between the microbiome and radiotherapy and promises to serve as both biomarker and therapeutic target in combatting CRC.
